# Trends in Preventable Hospitalization Rates for Children With or Without Observation Stay Data

**DOI:** 10.1001/jamanetworkopen.2025.1533

**Published:** 2025-03-24

**Authors:** Yao Tian, Michelle L. Macy, Jason M. Hockenberry, Jane L. Holl, Amber K. Sabbatini, Ronald T. Ackermann, Kristin Kan, Lynn Huang, Mehul V. Raval

**Affiliations:** 1 Department of Surgery, Northwestern University Feinberg School of Medicine, Chicago, Illinois; 2 Center for Health Services and Outcomes Research, Institute for Public Health and Medicine, Feinberg School of Medicine, Northwestern University, Chicago, Illinois; 3 Department of Pediatrics, Northwestern University Feinberg School of Medicine, Chicago, Illinois; 4 Mary Ann and J. Milburn Smith Child Health Outcomes, Research, and Evaluation Center, Ann and Robert H. Lurie Children’s Hospital of Chicago, Chicago, Illinois; 5 Division of Emergency Medicine, Ann and Robert H. Lurie Children’s Hospital of Chicago, Chicago, Illinois; 6 Department of Health Policy and Management, Yale School of Public Health, New Haven, Connecticut; 7 Department of Neurology, Biological Sciences Division and Center for Healthcare Delivery Science and Innovation, University of Chicago, Chicago, Illinois; 8 Department of Emergency Medicine, University of Washington School of Medicine, Seattle; 9 Department of Health Systems and Population Health, University of Washington School of Public Health, Seattle, Washington; 10 Department of Medicine, Northwestern University Feinberg School of Medicine, Chicago, Illinois; 11 Institute for Public Health and Medicine, Northwestern University Feinberg School of Medicine, Chicago, Illinois; 12 Division of Pediatric Surgery, Department of Surgery, Northwestern University Feinberg School of Medicine, Ann and Robert H. Lurie Children’s Hospital of Chicago, Chicago, Illinois

## Abstract

**Importance:**

Potentially preventable hospitalization rates are widely endorsed quality indicators that have been used to evaluate the impact of policies on access to high-quality primary and ambulatory care for children. Although potentially preventable hospitalization rates have been decreasing, little is known about how these trends are associated with the exclusive reliance on inpatient data, which omit observation status, a common designation for pediatric hospital stays.

**Objective:**

To assess the implications of omitting observation stay data on potentially preventable hospitalization rate calculations over time.

**Design, Setting, and Participants:**

In this retrospective cross-sectional study, inpatient and observation stays for patients aged 6 to 17 years related to pediatric ambulatory care sensitive conditions (ACSCs), were identified from the Healthcare Cost and Utilization Project state-specific databases from Georgia, Iowa, Maryland, Nebraska, and Vermont, 2010 to 2019, and from Wisconsin, 2012 to 2019. The units of analysis include an overall aggregate level, state level, and county level. Data were analyzed from February to November 2024.

**Main Outcome and Measures:**

The main outcome was the pediatric quality indicator overall composite of potentially preventable hospitalization rates (hereafter the composite hospitalization rate) and was calculated using inpatient-only and combined (ie, inpatient and observation) hospital stay data per 100 000 children.

**Results:**

Among a total of 64 846 identified hospital stays (median [IQR] age, 10 [8-14] years for inpatient stays and 10 [7-13] for observation stays; 32 733 [50.5%] male; 573 [0.9%] Asian or Pacific Islander, 20 042 [30.9%] Black, 3413 [5.3%] Hispanic, 168 [0.3%] American Indian or Alaska Native, 22 970 [35.4%] White, and 1842 [2.8%] Other), 22 275 (34.4%) were observation stays. The use of observation stays grew from 2090 of 6923 stays (30.2%) in 2010 to 2525 of 5531 stays (45.7%) in 2019. When using inpatient-only data, the overall aggregate-level composite hospitalization rate per 100 000 children was 141.7 in 2010 and decreased to 71.0 in 2019, and the annual percentage change was −6.8% (95% CI, −6.8% to −6.8%; *P* < .001). When using combined data, the composite hospitalization rate was 203.0 in 2010 and 130.7 in 2019, respectively, with an annual percentage change of −4.5% (95% CI, −4.5% to −4.5%; *P* < .001). Most state-level and county-level composite hospitalization rates exhibited declining trends when using inpatient-only data. After combining observation stay data, the declines were attenuated or even reversed in some cases.

**Conclusion and Relevance:**

Decreases in the composite hospitalization rate for pediatric ACSCs were associated with the shifting of patient status from inpatient to observation stays. These findings emphasize the need for standardized reporting and inclusion of observation stay data to support disease surveillance, policy evaluation, and decision-making.

## Key Points

**Question:**

Are the decreasing trends in potentially preventable hospitalization rates for children a result of improved access to care or associated with the patient status shifting from inpatient to observation stays?

**Findings:**

In this cross-sectional study of 64 846 hospital stays, when omitting observation stay data, a decreasing trend in the hospitalization rate was found. This trend was attenuated or even reversed in some cases after including observation stays.

**Meaning:**

These findings emphasize the need for standardized reporting and inclusion of observation stay data to support disease surveillance, policy evaluation, and decision-making.

## Teaser

This cross-sectional study evaluates the implications of omitting observation stay data from potentially preventable hospitalization rate calculations for children over time.

## Introduction

Potentially preventable hospitalization rates are widely used quality indicators that have been used to evaluate the impact of policies and programs aiming to improve access to high-quality primary and ambulatory care for children.^1,2,3,4,5,6,7^ For example, promising findings from the recent California Medicaid expansion suggested reduced hospitalization rates and improvements in access to primary care for children.^2^ The US Centers for Disease Control and Prevention (CDC) is currently tracking emergency department (ED) visit and hospitalization rates to evaluate a multistate program that aims to control childhood asthma and reduce exacerbations.^5^

At the same time, studies have reported a patient status shifting from inpatient to observation stays for children who have a short-term hospitalization over the past decade.^8,9,10^ Yet the observation stay data are currently excluded from the potentially preventable hospitalization rate measures.^11^ In turn, this growing portion of pediatric hospital stays are not considered in current policy evaluations or payment reforms.^1,2,3,4,5,6^

The area-level pediatric quality indicators (PDIs) is a set of quality indicators that focus on potentially preventable hospitalizations among pediatric patients, developed and endorsed by the Agency for Healthcare Research and Quality (AHRQ).^12^ In this study, we leveraged the PDI overall composite (ie, PDI 90), a composite measure encompassing all area-level PDIs, and assessed the implications of omitting observation stay data on the PDI 90 calculation over time.

## Methods

### Data

This retrospective study used AHRQ’s Healthcare Cost and Utilization Project (HCUP) state-specific databases, including State Inpatient Databases (SID), State Ambulatory Surgery and Services Databases (SASD), and State Emergency Department Databases (SEDD), from Georgia, Iowa, Maryland, Nebraska, and Vermont, 2010 to 2019, and from Wisconsin, 2012 to 2019.^13^ The states and years were chosen based on the availability and completeness of inpatient and observation stay data.^14^ The units of analysis include an overall aggregate level, state level, and county level.^15^ This study was deemed nonhuman participant research by the Northwestern University institutional review board, so no informed consent was required. We followed the Strengthening the Reporting of Observational Studies in Epidemiology (STROBE) reporting guideline.

### Study Populations

To have a consistent measurement, we followed the AHRQ PDI 90 specification to define and identify the study populations.^16^ Specifically, this area-level PDI is a composite measure of potentially preventable hospitalizations for pediatric (aged 6-17 years) ambulatory care sensitive conditions (ACSCs) per 100 000 population: asthma, diabetes with short-term complications, gastroenteritis, or urinary tract infection. The assumption is that hospital stays related to these 4 ACSCs might be avoidable if patients have access to high-quality primary and ambulatory care.^12^ The specification also details exclusion criteria, such as encounters with a principal diagnosis code assigned to pregnancy, childbirth, and the puerperium.^16^ In addition, hospital stays without a valid county Federal Information Processing System code of patient residence or with a residence out of the corresponding state were excluded, as this precluded calculating PDI 90 at a specific geographic level. Outpatient visits without evidence of observation stay services were also excluded. Detailed identification of the observation stay services is described in the subsequent section.

### Identification of Observation and Inpatient Stays

Observation stays were identified using an existing indicator, HCUP_OS, from the HCUP SEDD and SASD databases.^17^ This indicator reflects evidence of an observation stay based on the presence of observation stay revenue codes, observation stay *Current Procedural Terminology* codes, and positive observation stay charge when revenue center codes are not available.^17^ When a patient has an observation stay in the ED and then is discharged, the encounter is identified as an observation stay in SEDD. When a patient has an observation stay in other outpatient settings, such as a surgical procedure and/or a diagnostic test, and then is discharged (ie, without admission to an inpatient stay), the stay is identified as an observation stay in SASD. Observation stays were composed of hospital stays documented with observation stay services in SEDD and SASD. There are some duplicate records in the combined SEDD and SASD. We unduplicated the combined data by excluding duplicate records in SASD using the HCUP unique record identifier, KEY.^14^ Inpatient stays were identified from the HCUP SID databases, which include inpatient discharges in that state regardless of payer.^13^ If a patient has an observation stay and then is admitted to the same hospital for an inpatient stay, the hospital stay is only included in the SID database (ie, an inpatient stay).^13^

### Study Outcomes

The key study outcome was PDI 90, the PDI overall composite of potentially preventable hospitalization rates (hereafter the composite hospitalization rate) across all 4 ACSCs. It was calculated using inpatient-only and combined (ie, inpatient and observation) hospital stay data per 100 000 children per year.^16^ Given a specific geographic area and a year, the denominator was the number of children aged 6 to 17 years that was retrieved from the US Census Bureau and directly downloaded from AHRQ Quality Indicators software.^18^ The numerator for the inpatient-only hospitalization rate was the sum of potentially preventable inpatient stays; the numerator for the combined (inpatient and observation) rate was the sum of potentially preventable inpatient and observation stays, given the corresponding geographic area and year.^16^ Both the denominator and numerator were calculated at overall aggregate, state, and county levels. We also calculated the proportion of observation stays out of all hospital stays to assess the trends in observation stays over years at the overall and state levels.

### Other Measures

Demographic characteristics included sex, age, race and ethnicity, health insurance status, and metropolitan statistical area (MSA) categories of patient residence (ie, large MSA, small MSA, and non-MSA). The race and ethnicity data were extracted from the HCUP state-specific administrative databases. Recognizing that race and ethnicity are a social, and not biological variable, we included this information as racial disparities in access to care is an important factor to consider in this analysis. Year of discharge, an existing and ubiquitously coded variable, was used to model a time trend.^19,20^ Length of stay (LOS), a standardized data element in HCUP state databases, is calculated by subtracting the admission date from the discharge date.^19,20^ Furthermore, we categorized LOS for each observation stay using a categorical variable with 4 groups: 0 days, 1 day, 2 days, and more than 2 days, as most payers defined an observation stay using an expected LOS less than 24 (1 day) or 48 (2 days) hours.^21,22,23,24,25,26^

### Statistical Analysis

Data analysis was conducted from February 1 through November 30, 2024. Descriptive statistics were grouped by inpatient and observation status and were summarized using frequency, percentage, and quartiles (the first quartile [Q1], the second quartile [median], and the third quartile [Q3]). χ^2^ or Wilcoxon rank-sum tests were performed to examine differences between observation and inpatient stays.

We assumed that the proportion of observation stays was approximately normally distributed, and we also conducted the Shapiro-Wilk and Kolmogorov-Smirnov tests to examine this assumption.^27^ Furthermore, we performed a first-order autoregressive model to estimate the overall trend in use of observation stay data and the trend by states. We also assumed that the key outcome, PDI 90, followed a Poisson distribution and used a Poisson regression to estimate the percentage change of the composite hospitalization rate over years while accounting for repeated measures over years using generalized estimating equations. The exponentiated estimated coefficient of year from Poisson regressions can be interpreted as a percentage change in the rate.^28^ Furthermore, for the county-level trends, we conducted stratified analyses by MSA categories and included an interaction term between state and year.

All analyses were performed using SAS software, version 9.4 (SAS Institute Inc). A 2-sided *P* < .05 was considered statistically significant, and 95% CIs were presented.

## Results

Over the study period, a total of 64 846 hospital stays (median [IQR] age, 10 [8-14] years for inpatient stays and 10 [7-13] for observation stays; 32 733 [50.5%] male; 573 [0.9%] Asian or Pacific Islander, 20 042 [30.9%] Black, 3413 [5.3%] Hispanic, 168 [0.3%] American Indian or Alaska Native, 22 970 [35.4%] White, and 1842 [2.8%] Other) were identified, of which 22 275 (34.4%) were observation stays (Table 1). Race and ethnicity information was not available for 15 838 hospital stays. Among these observation stays, 16 805 (75.4%) and 5470 (24.6%) were found in SEDD and SASD, respectively. Most patients had public health insurance (36 426 patients [56.2%]). Among a total of 30 161 stays for patients residing in a large MSA, 8419 (27.9%) were observations stay, whereas for the remaining 34 685 stays of patients not residing in large MSAs, 13 856 (40.0%) were observation stays. The most common reason for both inpatient and observation stays was asthma. A total of 2083 (17.4%) of them were documented in SASD. The median (IQR) LOS was 1 (1-2) and 2 (1-3) days for observation and inpatient stays, respectively.

**Table 1.** Patient Characteristics of This Study

Characteristic	Patients, No. (%)		*P* value
	Inpatient stays (n = 42 571)	Observation stays (n = 22 275)	
Age, median (IQR), y	10 (8-14)	10 (7-13)	<.001
Sex			
Female	21 342 (50.13)	10 771 (48.35)	<.001
Male	21 229 (49.87)	11 504 (51.65)	
Race and ethnicity^a^			
Asian or Pacific Islander	377 (1.18)	196 (1.15)	<.001
Black	14 009 (43.91)	6033 (35.27)	
Hispanic	2018 (6.33)	1395 (8.16)	
Native American	122 (0.38)	46 (0.27)	
White	14 255 (44.68)	8715 (50.95)	
Other	1123 (3.52)	719 (4.20)	
Health insurance^a^			
Public	23 807 (56.10)	12 619 (56.82)	<.001
Private	16 420 (38.69)	8287 (37.31)	
Self-pay	1061 (2.50)	713 (3.21)	
Other	1151 (2.71)	591 (2.66)	
Urban-rural designation for the patient’s county of residence			
Large MSA (1 million residents or more)	21 742 (51.07)	8419 (37.80)	<.001
Small MSA	12 676 (29.78)	7787 (34.96)	
Non-MSA	8153 (19.15)	6069 (27.25)	
Conditions			
Asthma	22 471 (52.78)	11 943 (53.62)	<.001
Diabetes with short-term complications	9586 (22.52)	1127 (5.06)	
Gastroenteritis	5062 (11.89)	7135 (32.03)	
Urinary tract infection	5452 (12.81)	2070 (9.29)	
State			
Georgia	18 011 (42.31)	10 930 (49.07)	<.001
Iowa	4555 (10.70)	2987 (13.41)	
Maryland	11 402 (26.78)	3968 (17.81)	
Nebraska	3115 (7.32)	2056 (9.23)	
Vermont	404 (0.95)	218 (0.98)	
Wisconsin	5084 (11.94)	2116 (9.50)	

Abbreviation: MSA, metropolitan statistical area.

^a^ Records with missing values were not included in the descriptive statistics.

**Figure 1.**
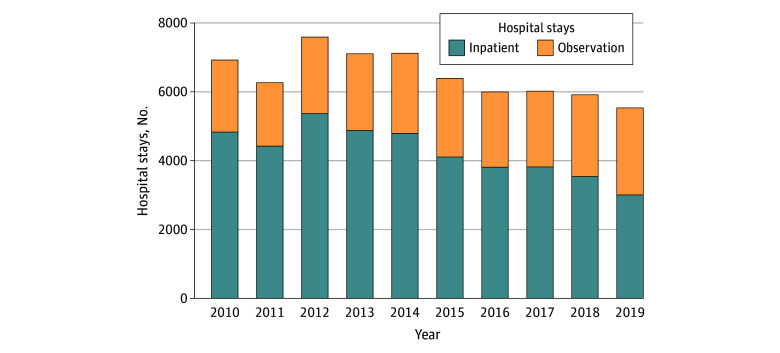
Trends in Potentially Preventable Hospitalizations by Stay Status Data were retrieved from Georgia, Iowa, Maryland, Nebraska, and Vermont, 2010 to 2019, and from Wisconsin, 2012 to 2019.

**Figure 2.**
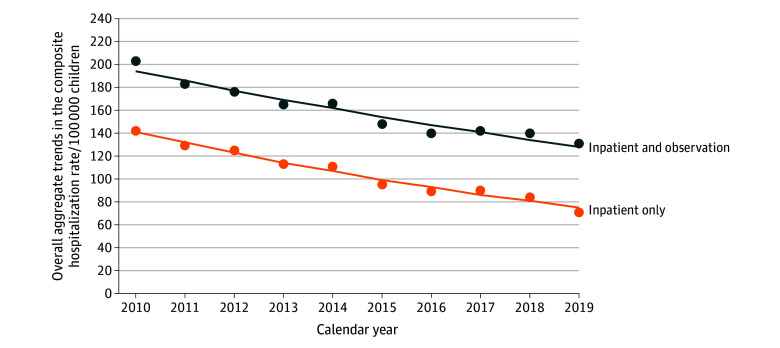
Overall Aggregate Trends in the Pediatric Quality Indicator Overall Composite of Potentially Preventable Hospitalization Rates, per 100 000 Children, With or Without Observation Stay Data Empirical values are presented along with predicated values (lines).

As shown in Figure 1, the proportion of observation stays increased from 30.2% (2090 of 6923 stays) in 2010 to 45.7% (2525 of 5531 stays) in 2019, with a yearly slope of 1.7% (95% CI, 0.7% to 2.7%). The proportion with an LOS of 2 days increased from 23.1% (480 of 2081 stays) in 2010 to 29.4% (743 of 2525 stays) in 2019, and the proportion with an LOS greater than 2 days increased from 2.3% (48 of 2081 stays) to 4.8% (122 of 2525 stays) (eFigure 1 in Supplement 1). When omitting observation stay data, the overall aggregate-level composite hospitalization rate (per 100 000 children) was 141.7 in 2010 and decreased to 71.0 in 2019 (Figure 2), and the annual percentage change was −6.8% (95% CI, −6.8% to −6.8%; *P* < .001). After including the observation stay data, the composite hospitalization rate was 203.0 in 2010 and 130.7 in 2019, respectively, with an annual percentage change of −4.5% (95% CI, −4.5% to −4.5%; *P* < .001).

At the state level, observation stays increased significantly in Iowa, Maryland, Nebraska, and Wisconsin (eTable 1 and eTable 2 in Supplement 1). In particular, the proportion of observation stays in Wisconsin significantly increased from 13.2% (133 of 1009 stays) in 2012 to 42.6% (366 of 859 stays) in 2019 with a yearly slope of 4.8% (95% CI, 1.9% to 7.6%); whereas the proportion in Vermont was 35.4% (29 of 82 stays) in 2010 and fairly stable during the same time period with a yearly slope of −0.01% (95% CI, −1.76% to 1.75%). The proportion of observation stays was 45.4% (531 of 1171 stays) in 2010 and 52.8% (198 of 375 stays) in 2019 for patients residing in non-MSAs of Georgia, whereas the proportion was 25.3% (315 of 1245 stays) in 2010 and 48.7% (718 of 1474 stays) in 2019 for patients residing in the large MSA of Georgia. Most state-level composite hospitalization rates exhibited trends that were aligned with the overall trend (Table 2; eFigure 2 in Supplement 1).

**Table 2.** The Annual Percentage Change of the State-Level Composite Hospitalization Rate Using Inpatient-Only and Combined Data From 2010 Through 2019^a^

State	% Change (95% CI)	
	Inpatient-only	Combined data (inpatient and observation)
Georgia	−4.1 (−4.5 to −3.7)	−3.6 (−3.6 to −3.6)
Iowa	−7.1 (−7.1 to −7.1)	−5.0 (−5.0 to −5.0)
Maryland	−8.6 (−8.6 to −8.6)	−4.4 (−4.4 to −4.4)
Nebraska	−7.0 (−7.1 to −6.9)	−4.2 (−4.2 to −4.2)
Vermont	−4.9 (−4.9 to −4.9)	−4.8 (−4.8 to −4.8)
Wisconsin	−9.2 (−10.5 to −7.8)	−2.1 (−2.1 to −2.1)

^a^ Because of large sample sizes of denominators, some of the 95% CIs are identical to the point estimate.

At the county level, we found variation in the trend of the composite hospitalization rate among various MSA categories (Table 3). For counties located in large MSAs of Maryland and Wisconsin, a decreasing trend in the rate was found when using inpatient-only data, with a percentage change of −9.4% (95% CI, −12.2% to −6.4%) for Maryland and −11.0% (95% CI, −13.1% to −8.8%) for Wisconsin. After including observation stay data, the decreasing trend was attenuated, with a percentage change of −5.2% (95% CI, −7.1% to −3.3%) and −1.8% (95% CI, −3.0% to −0.7%) for Maryland and Wisconsin, respectively. In contrast, for counties located in large MSAs of Georgia, the percentage change in the composite hospitalization rate declined when using inpatient-only data, with a percentage change of −2.7% (95% CI, −4.3% to −1.1%), yet increased after combining observation stay data, with a percentage change of 1.3% (95% CI, 0.1% to 2.5%). For counties located in small MSAs of Maryland and Wisconsin, the percentage change in the rate was −11.2% (95% CI, −19.0% to −2.6%) and −3.8% (95% CI, −6.1% to −1.5%) when using inpatient-only data, and after combining observation stay data, no significant trends were found. In Georgia, Iowa, and Nebraska, significantly decreasing trends in the rate were found, with and without observation stay data. In Vermont, increasing trends in the composite hospitalization rate were detected, with percentage changes of 5.4% (95% CI, 2.5% to 8.3%) and 2.2% (95% CI, −2.5% to 7.1%), with and without observation stay data, respectively. For all counties located in non-MSAs, we found decreasing trends in the composite hospitalization rate when using inpatient-only data and attenuation of the decreasing trend after combining observation stay data (Table 3).

**Table 3.** The Annual Percentage Change of the County-Level Composite Hospitalization Rate, Using Inpatient-Only and Combined Data From 2010 Through 2019

State	% Change (95% CI)	
	Inpatient-only	Combined data (inpatient and observation)
Large MSA		
Georgia	−2.7 (−4.3 to −1.1)	1.3 (0.1 to 2.5)
Maryland	−9.4 (−12.2 to −6.4)	−5.2 (−7.1 to −3.3)
Wisconsin	−11.0 (−13.1 to −8.8)	−1.8 (−3.0 to −0.7)
Small MSA		
Georgia	−4.3 (−6.1 to −2.6)	−5.1 (−7.4 to −2.9)
Iowa	−5.1 (−7.4 to −2.7)	−4.2 (−6.4 to −1.9)
Maryland	−11.2 (−19.0 to −2.6)	−3.5 (−9.7 to 3.1)
Nebraska	−4.7 (−7.2 to −2.2)	−2.9 (−4.6 to −1.2)
Vermont	5.4 (2.5 to 8.3)	2.2 (−2.5 to 7.1)
Wisconsin	−3.8 (−6.1 to −1.5)	−0.4 (−2.6 to 1.8)
Non-MSA		
Georgia	−11.6 (−14.1 to −9.1)	−10.5 (−12.6 to −8.4)
Iowa	−9.9 (−12.1 to −7.6)	−5.8 (−7.4 to −4.2)
Maryland	−6.8 (−11.1 to −2.3)	−2.1 (−9.4 to 5.7)
Nebraska	−8.1 (−11.1 to −5.1)	−5.2 (−7.2 to −3.2)
Vermont	−11.0 (−20.3 to −0.6)	−9.9 (−16.4 to −2.9)
Wisconsin	−7.4 (−10.3 to −4.3)	−4.7 (−6.9 to −2.4)

Abbreviation: MSA, metropolitan statistical area.

## Discussion

When using inpatient-only data, we found considerable declines in the composite hospitalization rate in all 6 states, which could be interpreted as a substantial improvement in access to high-quality primary and ambulatory care for children over the study years. However, after combining observation stay data, the decline was either attenuated or even reversed in some cases. Similar results have been reported in prior studies of the potentially preventable hospitalization rate for Medicare beneficiaries from 2010 through 2015^29,30^ but have not been shown in pediatric data.

Unlike the universal 2 midnight rule for Medicare populations,^31^ policies for pediatric populations vary by states and payers and have evolved over time. This study showed that the proportion of observation stays in Wisconsin significantly increased from 13.2% in 2012 to 42.6% 2019, whereas the proportion in Vermont was 35.4% in 2010 and fairly stable over the study years. Currently, several State Children’s Health Insurance Programs (CHIP) and commercial payers have either adopted the 2 midnight rule or developed a similar policy (eg, 24 or 72 hours) to assign observation status to a child’s hospital stay.^21,22,23,24,25,26^ For example, the BadgerCare Plus (ie, Medicaid/CHIP program in Wisconsin) allows for an observation stay of up to 72 hours,^21^ whereas the Maryland Department of Health denies the entire claim if a hospital bills more than 24 hours on a single observation stay.^24^ The state-level variation observed in this study may be attributed in part to when each state initiated their observation stay policy and its evolution over time. To better understand the variation and its impact on quality indicator calculation, official and publicly available documentation of the state-level policies as well as implementation dates would be helpful.

This study found variation in the trend of county-level composite hospitalization rate over years among various MSA categories when comparing the trend with or without observation stay data. For patients residing in the large MSA of Georgia, a decreasing trend in the county-level composite hospitalization rate calculated using inpatient-only data was reversed after combining observation stay data. For patients residing in non-MSAs of Georgia, the trend in the county-level composite hospitalization rate was about the same when using inpatient-only and combined data. Several explanations are possible. The use of observation stays was already elevated for patients residing in rural areas in 2010, and the increase from 2010 through 2019 was merely more pronounced in urban compared with rural areas. Specifically, data from this study show that the proportion of observation stays was 45.4% in 2010 and 52.8% in 2019 for patients residing in non-MSAs of Georgia, whereas the proportion was 25.3% in 2010 and 48.7% in 2019 for patients residing in the large MSA of Georgia. As a result, the percentage change of the hospitalization rate in the large MSAs of Georgia was dramatically different after including observation stay data. Using Georgia ED visit data, the Georgia Department of Public Health found that asthma-related ED visit rates among children living in the large MSA of Georgia increased from 2010 to 2018.^32^ Furthermore, studies have shown that children with asthma are admitted for observation stays through EDs.^8,33^ These findings suggest that the asthma-related observation stay rate among children living in the large MSA of Georgia increased during the study period, which might also contribute to the variation discovered in this study.

We further found that observation stays composed 16 805 (75.4%) and 5470 (24.6%) encounters from SEDD and SASD, respectively. In particular, 17.4% of observation stays due to asthma were documented in SASD. Although the SASDs are named for their focus on ambulatory surgical procedures, they also include encounter-level data for various outpatient services such as observation stays and imaging. However, the reporting and inclusion of observation stays in the SASD and/or SEDD vary by state and data year.^13^ Omitting this observation stay data likely yields an underestimated assessment of the potentially preventable hospitalization rate. At present, the CDC National Asthma Control Program funds 25 partners to improve asthma control services, yet only ED visits and inpatient stays are currently being reported.^34,35,36,37,38^ Meanwhile, only ED visits and inpatient stays are being measured and reported when evaluating Medicaid payment reforms, such as a statewide accountable care organization.^6^ Our findings suggest that it is essential to standardize the reporting and inclusion of observation stay data to support disease surveillance, policy evaluation, and decision-making.

### Limitations

This study has limitations. Our findings cannot be generalized to other states in which the observation stay policy and its implication on the hospitalization rate might be different. Due to limited documentation of observation stay policy over time, we were not able to gauge the impact of a specific policy on the potentially preventable hospitalization rate. In addition, the results from Vermont should be considered and explained with caution, as we were not able to include patients who resided in the state but received care at a hospital located in another state. Additionally, administrative data used in this study do not provide granular clinical information. For example, the measure of LOS in the HCUP datasets is based on days, and we were not able to assess LOS with hours. Future studies should explore LOS using hours when the data are available. Also, the indicator of an observation stay used in this study, HCUP_OS, is coded based on billing codes. We were not able to verify whether each observation stay fully met clinical criteria for observation care.

## Conclusions

In this study, the overall aggregate-level hospitalization rate decreased between 2010 and 2019; however, there was substantial variation at the county level. Metrics such as potentially preventable hospitalization rates are being increasingly used to reflect access to high-quality primary and ambulatory care. Our findings suggest that it is essential to standardize the reporting and inclusion of observation stay data to support disease surveillance, policy evaluation, and decision-making. Meanwhile, researchers, practitioners, and policymakers who are currently using the potentially preventable hospitalization rates to evaluate access to primary and ambulatory care for pediatric populations should account for both inpatient and observation stay data.

## Notes

Supplement 1. **eTable 1.** The Number of Observation Stays and Their Proportion of the Total Hospital Stays Over Years by States **eTable 2.** A Yearly Slope Change in the Proportion of Observation Stays by States **eFigure 1.** The Trend in Length of Stay Among Observation Stays Over Years **eFigure 2.** Trends in Pediatric Quality Indicator Overall Composite of Potentially Preventable Hospitalization Rates, per 100 000 Children, With or Without Observation Stay Data, by State

Supplement 2.
Data Sharing Statement
