# Supplementary material for: Trends in Preventable Hospitalization Rates for Children With or Without Observation Stay Data
Source: JAMA Netw Open. 2025 Mar 24;8(3):e251533. doi: 10.1001/jamanetworkopen.2025.1533 (PMC11933988; doi:10.1001/jamanetworkopen.2025.1533)
Supplement: Supplement 1. — eTable 1. The Number of Observation Stays and Their Proportion of the Total Hospital Stays Over Years by States eTable 2. A Yearly Slope Change in the Proportion of Observation Stays by States eFigure 1. The Trend in Length of Stay Among Observation Stays Over Years eFigure 2. Trends in Pediatric Quality Indicator Overall Composite of Potentially Preventable Hospitalization Rates, per 100 000 Children, With or Without Observation Stay Data, by State [file jamanetwopen-e251533-s001.pdf]

Supplemental Online Content

Tian Y, Macy ML, Hockenberry JM, et al. Trends in preventable hospitalization rates for children with or without observation stay data. *JAMA Netw Open*. 2025;8(3):e251533. doi:10.1001/jamanetworkopen.2025.1533

eTable 1. The Number of Observation Stays and Their Proportion of the Total Hospital Stays Over Years by States

eTable 2. A Yearly Slope Change in the Proportion of Observation Stays by States

eFigure 1. The Trend in Length of Stay Among Observation Stays Over Years

eFigure 2. Trends in Pediatric Quality Indicator Overall Composite of Potentially Preventable Hospitalization Rates, per 100 000 Children, With or Without Observation Stay Data, by State

This supplemental material has been provided by the authors to give readers additional information about their work.

© 2025 Tian Y et al. *JAMA Network Open*.

eTable 1. The Number of Observation Stays and Their Proportion of the Total Hospital Stays Over Years by States §

	2010	2011	2012	2013	2014	2015	2016	2017	2018	2019
Georgia	1353 (38.32)	1091 (35.64)	1195 (36.88)	1222 (38.40)	1133 (39.16)	968 (37.55)	819 (31.12)	848 (32.59)	1099 (41.10)	1202 (47.17)
Iowa	322 (36.02)	291 (32.44)	315 (37.02)	324 (38.25)	325 (39.93)	331 (42.54)	258 (41.61)	272 (43.04)	265 (43.59)	284 (47.25)
Maryland	178 (9.88)	245 (14.96)	335 (18.83)	344 (21.2)	413 (24.14)	506 (32.29)	500 (34.79)	504 (34.47)	470 (36.24)	473 (44.83)
Nebraska	208 (33.82)	185 (32.17)	218 (34.28)	152 (35.43)	241 (40.44)	234 (43.98)	222 (49.55)	217 (46.17)	194 (42.92)	185 (44.26)
Vermont	29 (35.37)	31 (32.63)	25 (34.25)	17 (29.31)	22 (39.29)	22 (38.60)	23 (47.92)	17 (32.69)	17 (33.33)	15 (30)
Wisconsin	NA	NA	133 (13.18)	166 (17.18)	194 (18.48)	222 (25.34)	361 (44.68)	342 (42.75)	332 (39.9)	366 (42.61)

Abbreviation: NA-Not Available

eTable 2. A Yearly Slope Change in the Proportion of Observation Stays by States

State	Coefficient (95% CI), %
Georgia	0.66 (-1.48, 2.80)
Iowa	1.37 (1.07, 1.66)*
Maryland	3.58 (2.97, 4.19)*
Nebraska	1.45 (0.11, 2.80)*
Vermont	-0.01 (-1.76, 1.75)
Wisconsin	4.78 (1.93, 7.64)*

Abbreviation: CI- Confidence Interval
* p-value<0.05

© 2025 Tian Y et al. *JAMA Network Open*.

eFigure 1. The Trend in Length of Stay Among Observation Stays Over Years §

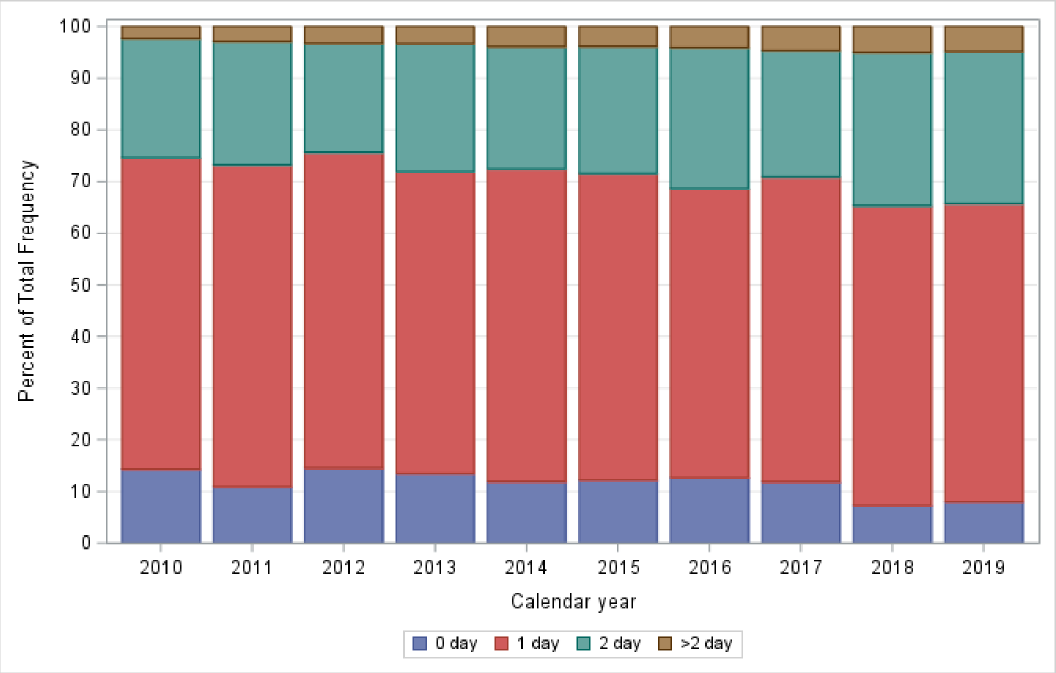

§ In this study, we categorized length of stay for each observation stay using a categorical variable with four groups: 0 day, 1 day, 2 days, and > 2 days, as most of payers defined an observation stay using an expected LOS less than 24 (1 day) or 48 hours (2 days).

© 2025 Tian Y et al. *JAMA Network Open*.

eFigure 2. Trends in Pediatric Quality Indicator Overall Composite of Potentially Preventable Hospitalization Rates, per 100 000 Children, With or Without Observation Stay Data, by State §

Figure 2(a) Georgia

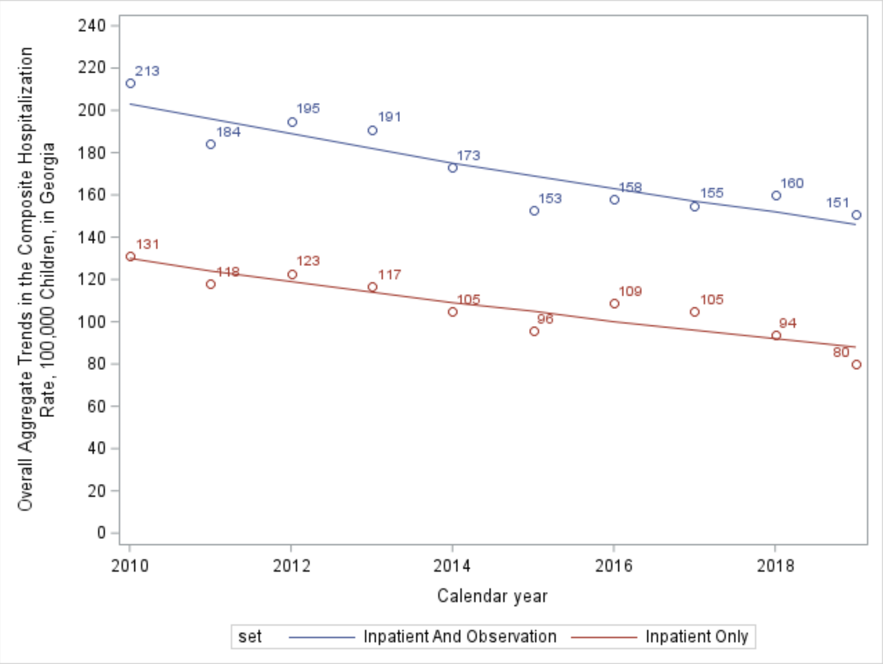

Figure 2(b) Iowa

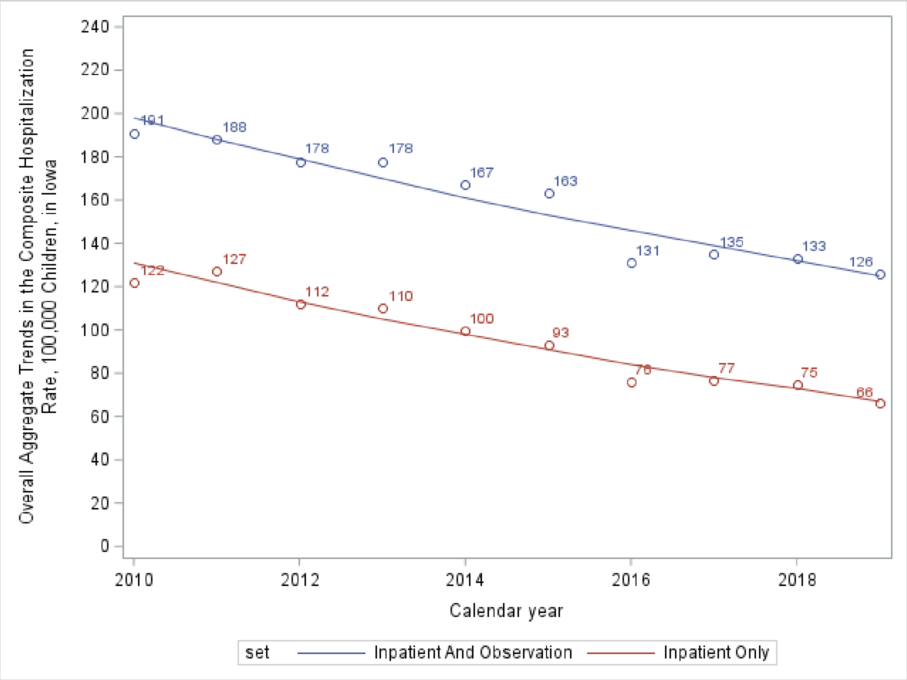

© 2025 Tian Y et al. *JAMA Network Open*.

Figure 2(c) Maryland

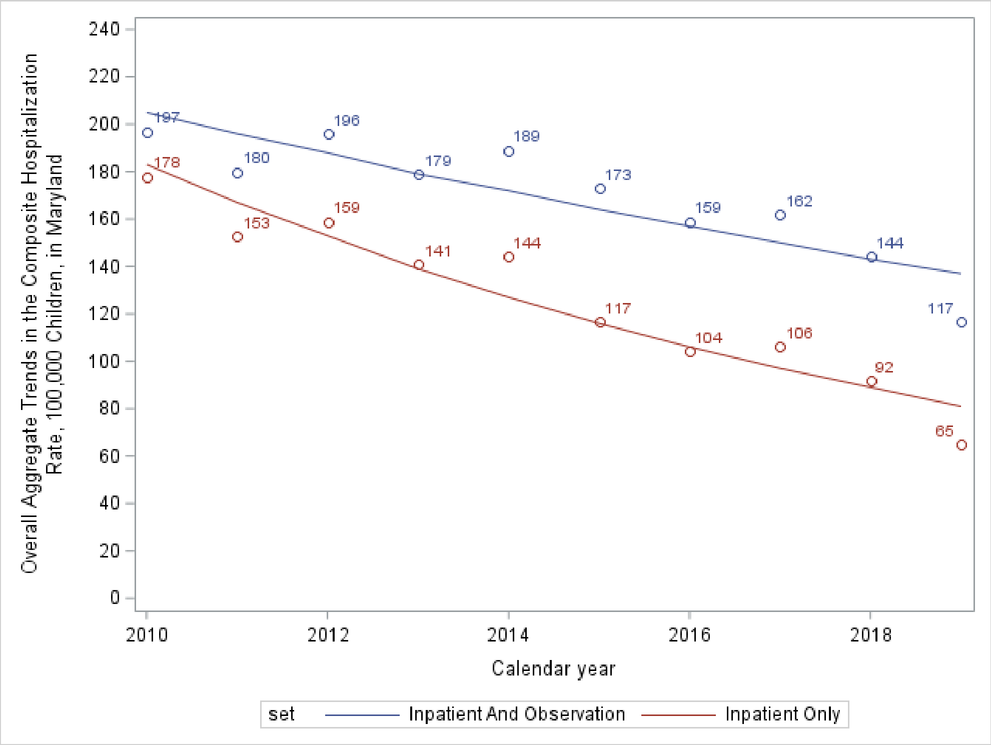

Figure 2(d) Nebraska

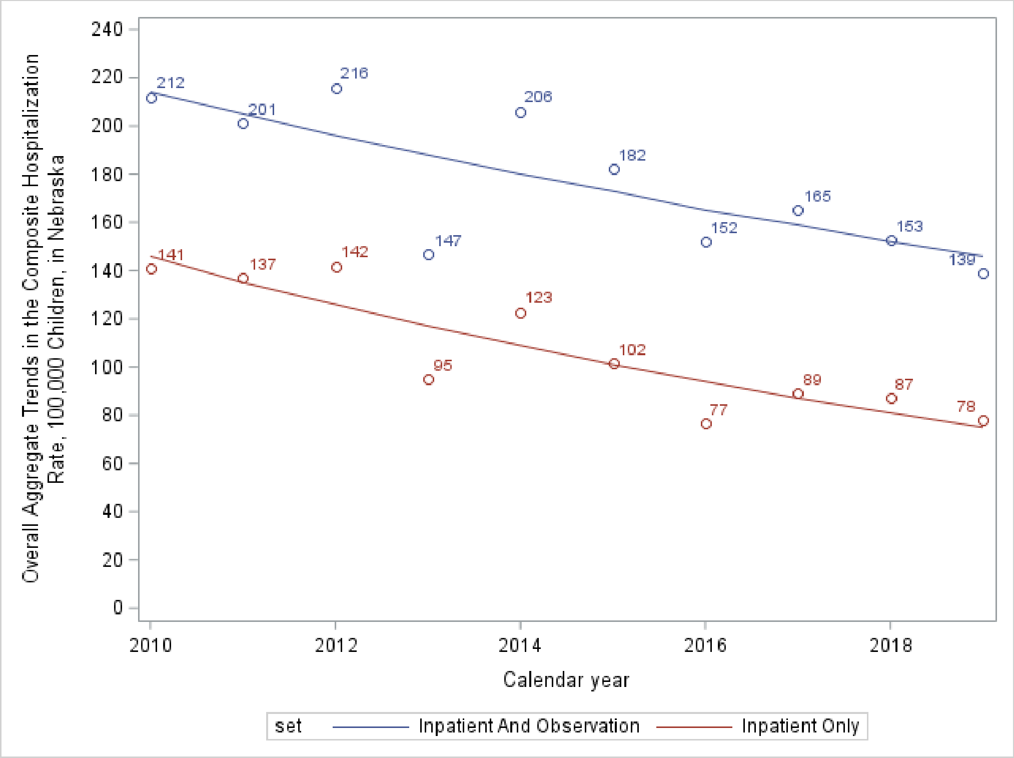

© 2025 Tian Y et al. *JAMA Network Open*.

Figure 2(e) Vermont

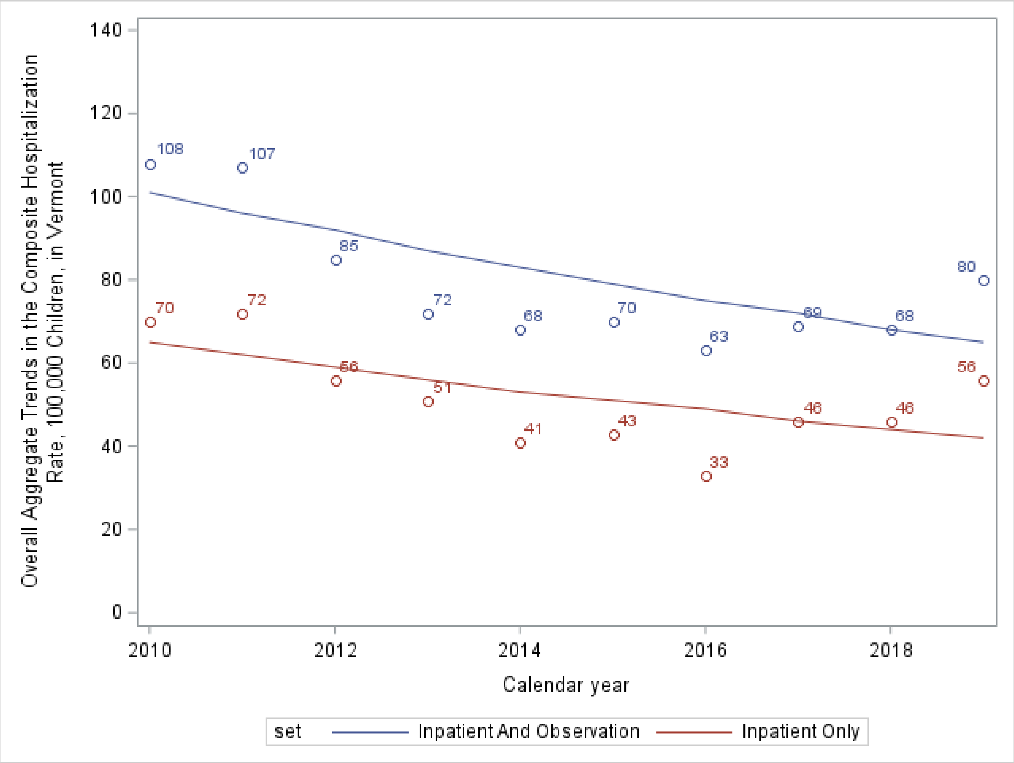

Figure 2(f) Wisconsin

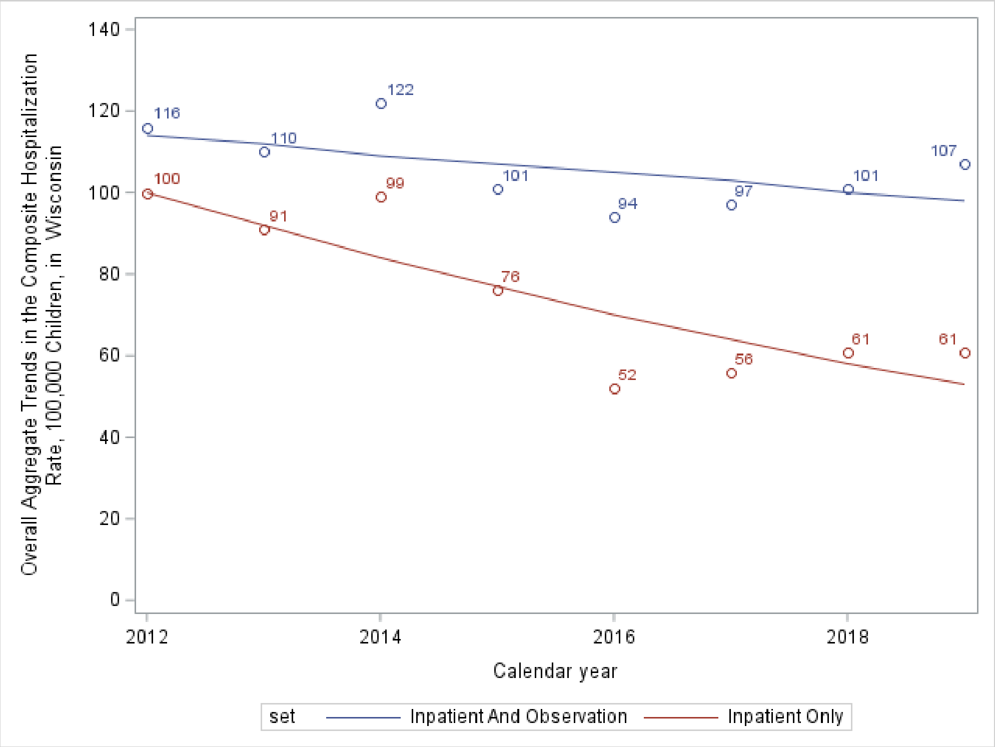

§ Empirical values are presented along with predicated values (lines)

© 2025 Tian Y et al. *JAMA Network Open*.
