# Supplementary material for: Trends in Preventable Hospitalization Rates for Children With or Without Observation Stay Data
Source: JAMA Netw Open. 2025 Mar 24;8(3):e251533. doi: 10.1001/jamanetworkopen.2025.1533 (PMC11933988; doi:10.1001/jamanetworkopen.2025.1533)
Supplement: Supplement 2. — Data Sharing Statement [file jamanetwopen-e251533-s002.pdf]

Data Sharing Statement

Tian. Trends in Preventable Hospitalization Rates for Children With or Without Observation Stay Data. *JAMA Netw Open*. Published March 24, 2025.
doi:10.1001/jamanetworkopen.2025.1533

Data
Data available: No

Additional Information
Explanation for why data not available: Data obtained from secondary data sources that require data use agreements for access and use.
